# Evaluation of CD44 and CD133 as cancer stem cell markers for colorectal cancer

**DOI:** 10.3892/or.2012.1951

**Published:** 2012-08-06

**Authors:** CHUNXIA WANG, JINGPING XIE, JIASONG GUO, H. CHARLES MANNING, JOHN C. GORE, NING GUO

**Affiliations:** 1 Department of Pharmacy, Nanfang Hospital, Southern Medical University, Guangzhou 510515, P.R. China; 2 Institute of Imaging Science, Vanderbilt University, Nashville, TN 37232, USA; 3 Department of Radiology and Radiological Sciences, Vanderbilt University, Nashville, TN 37232, USA; 4 Department of Embryology and Histology, Southern Medical University, Guangzhou 510515, P.R. China; 5 Department of Biomedical Engineering, Vanderbilt University, Nashville, TN 37232, USA; 6 Department of Electrical Engineering, Vanderbilt University, Nashville, TN 37232, USA; 7 Department of Physics and Astronomy, Vanderbilt University, Nashville, TN 37232, USA; 8 Department of Molecular Physiology and Biophysics, Vanderbilt University, Nashville, TN 37232, USA

**Keywords:** markers, cancer stem cells, colorectal cancer, apoptosis, proliferation, invasion

## Abstract

Colorectal cancer (CRC) is a major cause of cancer-related mortality in the world. Recently, a number of studies have demonstrated that cancer stem cells (CSCs) present in colorectal cancer tissues, are responsible for resistance to conventional therapies. Therefore, effective recognition of CSCs is of great importance. In the present study, to explore the potential characterizations of CSCs by the expression of specific cell surface markers such as CD133 and CD44, we screened six CRC cell lines using western blotting, immunofluorescence and flow cytometry. SW620, one of the six cell lines analyzed, was sorted into four subpopulations by fluorescence activated cell sorting (FACS). The capability of colony formation, proliferation rate, apoptosis, drug resistance, as well as their migratory and invasion potential were detected. The results revealed that the combination of CD44 and CD133 correlates with the features of CSCs in SW620 cells. CD44 positive cells showed more robust colony formation, higher proliferation, less spontaneous apoptosis, a higher resistance to drug-induced cell death, and were enriched after drug treatment. Among CD44 positive SW620 cells, the CD133 negative subpopulation was more migratory and invasive, which means that CD44^+^CD133^−^ correlates with most of features proposed for CSCs. Overall, the data presented herein showed that CRCs have a wide range of expression for CD44 and CD133; it is unlikely the CSCs can be characterized by any single marker or the same set of markers for all colon cancer cells. For SW620 cells, the CSCs are likely represented by the CD44^+^CD133^−^ surface marker. This finding of CSC markers represented by one positive and one negative is in line with CSCs in other tumors, such as CD34^+^CD38^−^ for acute myeloid leukemia; CD44^+^CD24^−^ for breast and pancreatic tumors. The absence of surface molecule(s) on CSCs will make it even more difficult to track and target this group of minority cells.

## Introduction

Colorectal cancer (CRC) represents a significant global health problem. It was estimated that 50% of people in the western world will develop a colorectal adenoma by the age of 50, with about one in ten patients progressing to malignancy (1). If be diagnosed at early stage, non-metastatic CRC can be surgically resected with a favorable prognosis. However, CRC metastases are associated with a poor prognosis and are often refractory to chemotherapy. Since CRC has a defined precursor cell population and exhibits slow progression to metastasis, a successful cancer treatment strategy will have to be based on the elimination of cancer stem cell (CSC) population. Thus, identifying CSCs is the crucial first step in the treatment of CRC.

CSCs were firstly found in acute myeloid leukemia (AML) and later in other tumor types. They have been identified through their expression of specific cell surface markers, such as CD34^+^CD38^−^ was associated with AML(2); CD44^+^CD24^−^ was for breast tumors(3); and CD44^+^CD24^−^ESA^+^ was for pancreatic tumors(4). One of the well documented CSC markers is CD133. CD133^+^ population is enriched in many tumor tissues including CRCs (5,6). On the other hand, since a single CD44^+^ CRC cell can form a sphere *in vitro* with stem cell features, and generate a xenograft tumor *in vivo* with the properties of the original tumor, CD44 was proposed as a robust marker for colon CSCs (7,8). In addition, CD44 was also reported as the marker for gastric cancer CSCs (9).

Another potential colon CSC marker is ALDH1, a detoxifying enzyme that oxidizes intracellular aldehydes and converts retinol to retinoic acid. Selection of CD133^+^, CD44^+^ cells with ALDH activity enriched somewhat the CSC population (10). However, either CD133 and CD44 or their combination can be used effectively as a marker for the identification of CSCs is still disputable (11). To assess whether CD44, CD133, or a combination of CD44 and CD133 can represent CSCs of CRC, we studied the expression pattern of popular markers on six CRC cell lines. Among them, SW620 cells were classified into four subpopulations based on the CD44 and CD133 expression. The capability of colony formation, proliferation, apoptosis, drug resistance, as well as the migratory and invasion potential of each subpopulation were subsequently analized. Our data suggested that CD44 and CD133 or their combination cannot universally be used to establish the identity of the CSCs for all CRCs, but CD44^+^CD133^−^ seems likely to represents the CSCs in SW620 cells.

## Materials and methods

### Cell lines and culture

Colon cancer cell lines, Colo205, DLD1, HCT116, HT29, SW480 and SW620 originated from the American Type Culture Collection (ATCC, Manassas, VA, USA), and cultured in DMEM containing 10% FBS supplemented with 100 IU/ml penicillin and 100 μg/ml streptomycin. All culture reagents were from Invitrogen (Carlsbad, CA, USA).

### Western blot analysis

Cells were lysed on ice by mammalian protein extraction reagent (ThermoFisher Scientific, Waltham, MA, USA) plus protease inhibitors (Sigma-Aldrich, St. Louis, MO, USA). After removing insoluble debris by centrifugation at 16,000 × g for 30 min at 4°C, the supernatant was designated as whole cell lysate. Protein concentrations were determined with Bradford method (Bio-Rad, Hercules, CA, USA). Protein (40 μg) for each cell lysate was separated by SDS-PAGE and transferred onto PVDF membranes (Bio-Rad). Membranes were blocked with 5% dry milk in TBST and immunoblotted with primary antibodies as follows: CD44, ESA (eBioscience, San Diego, CA, USA), CD133 (Miltenyi Biotec, Auburn, CA, USA) and ALDH1A1 (LifeSpan Biosciences, Seattle, WA, USA). β-tubulin antibody was used for loading control. HRP conjugated secondary antibodies (Jackson ImmunoResearch Laboratories, West Grove, PA, USA) and enhanced chemiluminescence (Pierce, Rockford, IL, USA) were used to detect the protein bands. Digital images of luminescence were taken by IVIS system (Caliper Life Sciences, Hopkinton, MA, USA).

### Immunofluorescence assay

Cells (1−10^3^) were planted onto 8-well glass chamber slides (Fisher Scientific, Hampton, NH, USA) and cultured for 24 h. After briefly rinsed with PBS twice, the cells were fixed with 4% paraformaldehyde for 30 min and washed with PBS three times. Then, the fixed cells were blocked with 10% normal goat serum plus 1% BSA (Sigma-Aldrich) for 30 min, and incubated with PE-conjugated CD133 (Miltenyi Biotec), FITC-conjugated CD44 and eFluor 660-conjugated ESA (eBioscience) for 1 h at 4°C in the dark. Subsequently, the slides were cover slipped with mounting medium (Dako) containing DAPI to counter stain the nuclei.

### Flow cytometry analysis and isolation of cell subpopulation

The expression profiles of CD133 and CD44 in cultured cells were analyzed by flow cytometry. Briefly, 1−10^6^ cells were incubated with 100 μl of 1% BSA in PBS containing 1 μg of CD16/CD32 (eBioscience) for 30 min on ice to block unspecific Fc interaction, then labeled with PE-conjugated anti-CD133, FITC-conjugated anti-CD44 and eFluor 660-conjugated anti-ESA antibodies for 1 h. Labeled cells were resuspended in PBS with 1% FBS, and analyzed by flow cytometer (BD Biotechnology). Isotypic IgG and unstained cells served as negative controls. By using the same setup, CD44 and CD133 co-labeled SW620 cells were sorted by FACS to obtain CD133^+^CD44^+^, CD133^+^CD44^−^, CD133^−^CD44^+^ and CD133^−^CD44^−^ subpopulations. Propidium iodide (1 μg/ml) (PI, Invitrogen) was added into the suspension of cells to exclude the dead cells during sorting.

### Colony-formation and cell proliferation assay

SW620, SW480 and their sorted cells were seeded to 96-well plates with an estimated single cell per well by limited dilution. Fourteen days later, the colonies were counted under phase contrast microscope. Cell proliferation rates were determined with CyQuant cell proliferation assay kit (Invitrogen). Cells (2,500) were seeded in 96-well plates with 200 μl growth medium per well. Cells were cultured for up to 5 days. At each selected time point started at 24 h, one plate was retrieved from incubator and stored at −70°C after the removal of culture medium. Once all the plates were collected, the cell numbers (total DNA) were quantitated by following the manufacturer’s protocol.

### Apoptosis assay based on caspase 3/7 activity

Aliquots of 1.5−10^4^ sorted cells were seeded on 96-well plates. After 24 h, 2 μM of camptothecin (CPT, Sigma-Aldrich) was added as treatment groups, medium with vehicle DMSO alone (Sigma) was also setup as the control group. After an additional 48 h of incubation, cellular apoptotic activity was assessed with caspase-Glo kit (Promega, Madison, WI) following the recommended procedures of the manufacturer. The fold increase in activity was calculated based on the normalized activity of untreated cells. The basal level activity of different subpopulation was also plotted. Each sample was measured three times.

### Tumor invasion and migration assay

These assays were performed by using BD BioCoat Tumor Invasion System. FluoroBlok 24-Multiwell Insert Plate with an 8 μm pore size PET membrane was used for migration assay and the same insert plate uniformly coated with BD Matrigel Matrix (BD Biosciences) was used for the invasion assay. Sorted SW620 cell subpopulations (1−10^6^) in 500 μl serum-free medium was seeded to the apical chamber, then 750 μl of chemoattractant (10% FBS in DMEM) was added to each of the basal chambers. After 48 hours incubation, cells migrated to the lower chamber were stained with 4 μg/ml calcein-AM in HBSS for 1 h at 37°C, 5% CO_2_. Fluorescence intensity was quantified by a bottom-reading plate reader. Insert membranes were also examined and fluorescent pictures were taken under an inverted fluorescence microscope.

### Statistical analysis

All of the data were analyzed with the GraphPad Prism (GraphPad Software, Inc., La Jolla, CA). Results are expressed as mean ± SD. Comparisons between two groups were assessed by two-tailed Student’s t-test. Differences between groups were considered significant with P<0.05. All experiments were performed at least twice to confirm reproducibility.

## Results

### Expression profiles of CD133, CD44, ALDH1 and ESA in selected colon cancer cell lines

**Figure 1:**
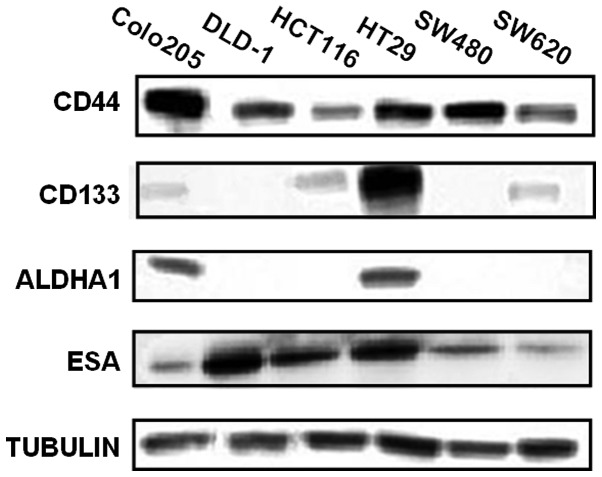
Western blot analysis for expression of CD133, CD44, ALDH1A1 and ESA. The cellular extract from six colon cancer cell types (40 μg of protein in each line) was separated on a 10% acrylamide gel. Expression levels of four markers were determined by using CD44, CD133, ESA and ALDH1A1 antibodies. Tubulin was used as loading control.

CD133, CD44, ESA and ALDH1 are widely considered as markers of cancer stem/progenitor-like cells. To test this hypothesis, western blotting was performed to analyze the expression profiles of these proteins in six colon cancer cell lines. As shown in Fig. 1, CD44 and ESA were detected in all tested cell lines with different expression levels; but CD133 was only detectable in Colo205, HCT116, HT29 and SW620; and ALDH1A1 only in Colo205 and HT29.

### Expression profiles of CD133 and CD44 by flow cytometric analysis

The relative percentages of cells expressing each single surface marker (CD133, CD44 and ESA) or multiple markers were determined by flow cytometric analysis. All the cells in the six cell lines were ESA positive (not shown). As shown in Table I, for single marker, ~94% of DLD1 cells were CD44 positive but only 12% were CD133 positive. By comparison, 69.6% of HCT116 cells were CD133 positive, but only 16.4% were CD44 positive. In contrast, almost all HT29 cells expressed these two surface markers. An interesting observation was from SW480 and SW620 lines which derived from the same patient, as the early stage cells, SW480 were almost all CD44 positive (95.8%), while only a very small percentage (0.2%) of cells were CD133 positive. In the later stage invasive SW620 cells, CD133 positive cells were around 60%, but only a small percentage (3.2%) was CD44 positive. These data suggest that ESA maybe a surface marker for colon tumor cells, but cannot be a CSC marker. Since the majority of cells express either CD133 or CD44 in many types of colon tumor cells, it is unlikely that a single marker of CD133 or CD44 can be used to distinguish CSCs.

**Figure 2:**
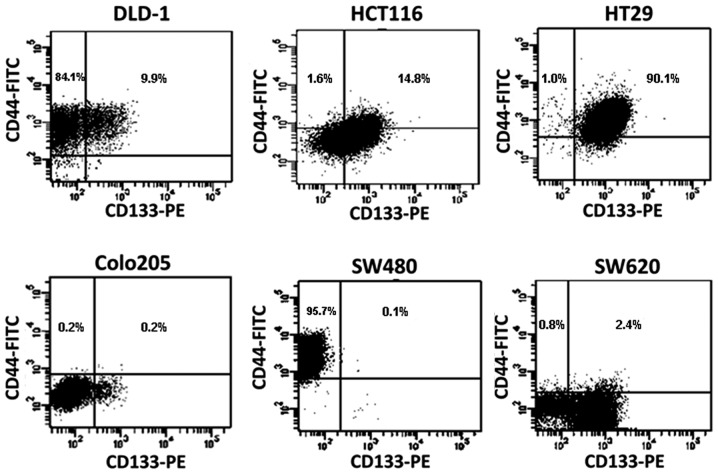
Expression profiles of CD133 and CD44 and their co-expression in Colo205, DLD1, HCT116, HT29, SW480 and SW620 cell lines characterized by flow cytometry. Suspensions cells were labeled with PE-conjugated anti-CD133 and FITC-conjugated anti-CD44 antibodies, and analyzed by a flow cytometer. Isotypic controls were used to establish the right gating. The top portion (two quarters) is CD44 positive; the left portion is CD133 positive. The double positive cells in the right top quarter are numbered as percentage of total cells.

In contrast to high percentage of cells expressing for single marker of CD44 or CD133, the percentage of cells with co-expression of CD44 and CD133 markers on the same cell is lower for most of cell lines, such as only 0.1% of SW480 cells, 0.2% of Colo205 cells, 2.4% of SW620 cells expressed CD133 and CD44 simultaneously. However, they are still high in DLD1 (9.9%), HCT116 (14.8%) and especially for HT29 (90.1%) cell lines (Fig. 2). Of note, the percentage of CD44^+^CD133^−^ cells is also very small in four of six cell lines, such as Colo205 (0.2%), HCT (1.6%), HT (1.0%) and SW620 (0.8%). These data suggested that the minority of the CSC subpopulation can be represented by the combination of CD133 and CD44 markers, not necessary the positive co-expression of both markers.

### Immunofluorescence assay

**Figure 3:**
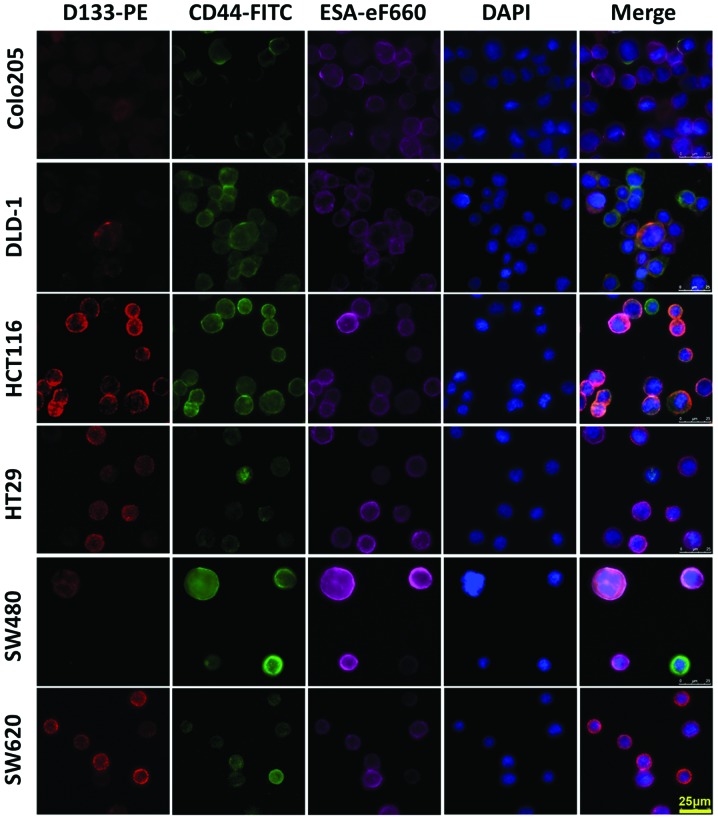
Immunofluorescence staining of CD133, CD44 and ESA in indicated cell lines. Cells were directly labeled using PE-conjugated CD133 (red), FITC-conjugated CD44 (green) and eFluor 660-conjugated ESA (purple) antibodies; nuclei were stained blue with DAPI.

To confirm the western blotting and flow cytometry data, cells labeled with PE-conjugated CD133 antibody (red) and FITC-conjugated CD44 antibody (green) were fixed, and the nuclei were counter-stained with DAPI (blue). These cell samples were examined under a fluorescence microscope. The fluorescent distribution pattern in individual cell line from the immunostaining with single antigen was consistent with the western blotting data. Overlapped multicolor images of triple-immunostaining comfirmed the existence of CD44^+^CD133^−^ and CD44^+^CD133^+^ cells (Fig. 3).

### Evaluation of colony formation and cell proliferation in different subpopulations

**Figure 4:**
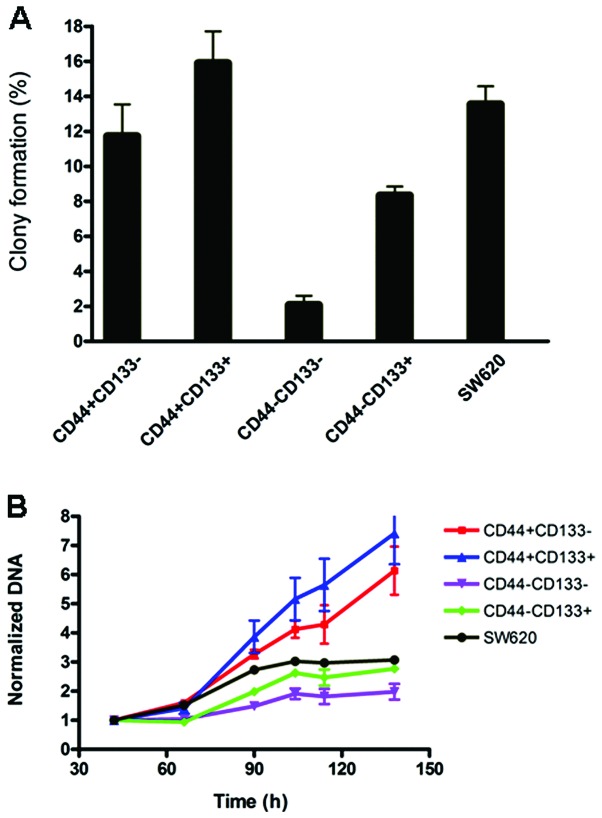
Capability of colony formation and proliferation analysis in sorted subpopulations of SW620 cells. SW620 cells sorted on their CD133 and CD44 reactivity were seeded by limited dilution as single cells into microtiter wells. After 14 days, the colonies formed were counted and calculated as percentage of total wells, initial colonies (A). (B) Cell proliferation analysis was based on DNA content. Growth curves show the proliferation rate of SW620-based cell lines and their subpopulations following seeding of 2500 cell density per 96-well at various time points. Data were normalized to their corresponding controls to compensate for minor differences in the initial cell counting and density.

To exam the properties of cell subpopulations expressing different surface markers, and explore whether the combination of CD44 and CD133 can be used to identify CSCs from certain colon cancer cells, SW620 cells were selected for further characterization. SW620 cells were sorted into CD44^+^CD133^+^, CD44^+^CD133^−^, CD44^−^CD133^+^ and CD44^−^CD133^−^ subpopulations by FACS. Then 200 μl of the suspension containing one single cell was planted into each well of 96-well plates. The number of colonies formed were counted after two weeks of culture. The results showed that CD44^+^CD133^+^ cells have the strongest clone forming capability among all subpopulations, and the CD44^−^CD133^−^ subpopulation produced the least clones (Fig. 4A). The data for unsorted control SW620 mixture were likely overestimated as compared to sorted subpopulations, since sorting would damage slightly the cells resulting in lower colony formation rate.

The relative proliferation rates of sorted cell subpopulations from the SW620 line were analyzed by measuring the total DNA content. As shown in Fig. 4B, all subpopulations only displayed slight differences in growth rate, CD133^+^ CD44^+^ cells had the fastest, and CD133^−^ CD44^−^ the slowest growth rate. CD44 expression seems correlated with growth rate by the comparison of the CD44^+^ and CD44^−^ cells.

### Spontaneous apoptosis and drug resistance

**Figure 5:**
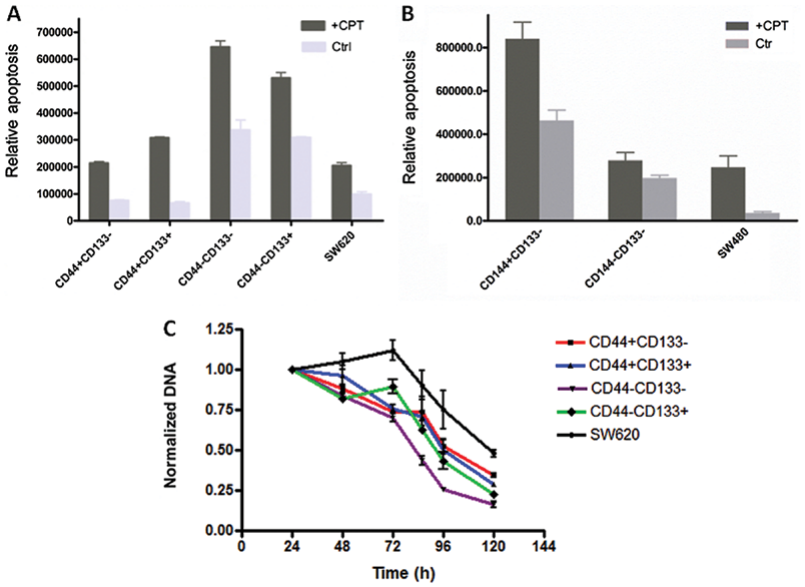
Camptothecin (CPT) was used to evaluate the resistance conferred by CD44 and CD133 to exogenous apoptotic stress. Each well of a 96-well plate was seeded with 15K sorted or unsorted cells. After 24 h, cells were treated with 2 μM CPT. (A and B) Apoptosis assays of SW620 and SW480 lines and their subpopulations 48 h after CPT treatment. (C) Cell growth curves in the presence of 2 μM CPT.

CD44^+^ cells from SW620 had the least spontaneous apoptosis, and were more resistant to the drug CPT (Fig. 5A). SW480 cells seemed to behave differently, a small portion of CD44 negative cells undergoing a less spontaneous apoptosis, even though they were more resistant to CPT, similar to the corresponding subpopulations of the SW620 line (Fig. 5B). Considering that the SW620 line is derived from the SW480 line, these data suggested either CD44 or CD133 alone has no direct correlation to their capability of drug resistance or to survive. It is also worth to note that unsorted cells of SW620 and SW480 lines showed a relatively low apoptosis rate, suggesting that cells with different markers support each other with respect to growth. To further test this, the proliferation profiles of sorted SW620 subpopulation cells were analyzed under the stress of drug treatment. As shown in Fig. 5C, the drug has the greatest impact on the growth of CD44^−^CD133^−^ cells, and the least on CD44^+^CD133^−^ and unsorted SW620 cells. These data are consistent with the results of the apoptotic assay.

**Figure 6:**
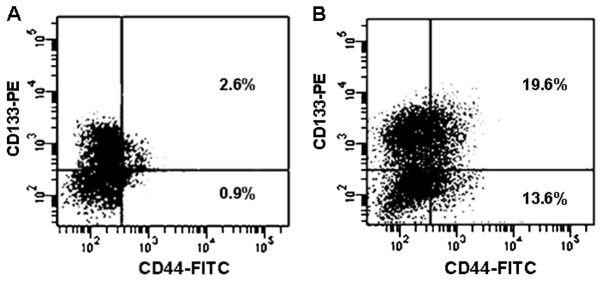
Effects of CPT treatment on the expression of CD133 and CD44. SW620 cells were treated with or without 2 μM CPT for 24 h, and then subjected to flow cytometric analysis of surface marker expression for both CD44 and CD133.

Next the changes of surface markers were examined for the viable cells after CPT treatment. As shown in Fig. 6, the percentage of CD44^+^ cells in the unsorted SW620 cells were increased by 24 h of treatment with 2 μM CPT; CD44^+^CD133^+^ cells showed increases from 2.6 to 19.6%. However, percentagewise, CD44^+^CD133^−^ increased the most from 0.9 to 13.6%.

### CD44^+^CD133^−^ subpopulation has the strongest invasion and migration capability

**Figure 7:**
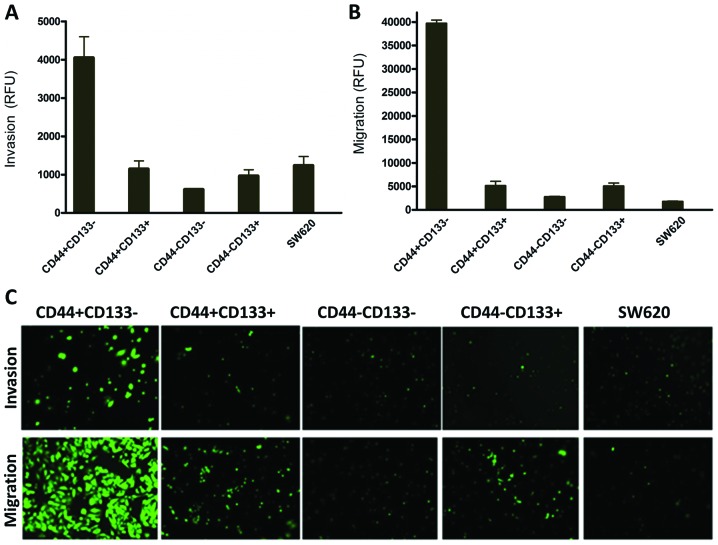
Assessments of migration and invasion potential of sorted SW620 cells by Matrigel. Sorted cells in serum-free medium was seeded into the apical chamber, chemoattractant was added to each of the basal chambers. After 48 h of incubation, cells that invaded through the Matrigel-coated or migrated through the non-coated membrane were stained with calcein-AM in HBSS. Fluorescence of migrated or invaded cells was quantified by a bottom-reading fluorescent plate reader. After subtracting controls, the fluorescence signal strength was plotted versus cell type. (A) Invasion assay; (B) migration assay; (C) inserted membranes were examined under a fluorescence microscope (magnification, −20).

The sorted SW620 cells with CD44^+^CD133^−^, CD44^+^CD133^+^, CD44^−^CD133^−^ and CD44^−^CD133^+^ phenotypes and unsorted SW620 cells were allowed to invade for 40 h using the BD Matrigel Invasion Chamber assay. As shown in Fig. 7A, CD44^+^CD133^−^ cells were 6-fold more invasive than CD44^−^CD133^−^ and 3-fold more than the unsorted cells. The CD44^−^CD133^−^ and CD44^−^CD133^+^ were weakly invasive and only scare spots of fluorescence were observed under the inverted fluorescent microscope (Fig. 7C). These data suggest CD44^+^CD133^−^ cells were the most invasive ones. Migration study conducted by BD Matrigel Chamber without coated membranes showed that the CD44-CD133- cells and unsorted cells were weakly migratory, while the CD44^+^CD133^−^ cells had the strongest migration capability; more precisely, CD44^+^CD133^−^ cells had a 14-fold higher migration rate than those of CD44^−^CD133^−^ cells as measured by fluorescence intensity (Fig. 7B). These data were confirmed by visual examination under an inverted fluorescent microscope (Fig. 7C).

## Discussion

CRC is the second leading cause of cancer-related mortality in developed countries. It is believed that cancers are developed from, and are maintained by CSCs arising from a resident normal stem/progenitor cell within the tissue bearing the characteristics of malignancy. A number of studies have demonstrated the existence of CSCs in CRC tissues (5,6). These CSCs have been characterized by their expression of specific cell surface biomarkers, such as CD133, CD44. CD44 is a transmembrane glycoprotein involved in cell adhesion, migration and drug resistance.

CD133 is a membrane protein originally classified as a marker of primitive hematopoietic and neural stem cells. CD133 was suggested to play a role in tumor angiogenesis as CD133^+^ glioma cells produce proangiogenic factors that can directly modify endothelial cell behavior (12). CD133 is recognized as a CSC marker in brain, colon, melanoma, bone sarcomas, non-small cell lung cancer, and other solid tumors (5,6,13–16), but this notion was challenged by studies from other groups. CD133 was found widely distributed in many epithelial tissues, and CD133 expression does not correlate with the ability of colon tumors to metastasize, as 40% human CRCs that metastasized to the liver are CD133 negative (11). CD133 expression can also be modulated by oxygen levels (17). Therefore, CD133 as CSC marker need to be re-evaluated and additional CSC surface markers maybe involved in maintaining CSC properties (18). To evaluate whether CD44, CD133 or combination of both can represent CSCs of CRC, we analyzed their protein expression levels in several CRC lines by western blotting.

As shown in Table I, CD44 and ESA are relatively highly expressed in all cell lines, suggesting that they are a common denominator but cannot be the sole CSC markers in CRCs. CD133 had the highest protein expression in HT29 cells; the percentage of cells expressing each marker is loosely related to total protein level. Such as HT29 had a higher CD133 protein level than HCT116 cells, the percentage of CD133 positive cells was also higher in HT29 than in HCT116 cells (98% vs. 70%). The total protein level of CD133 is similar in Colo205 and SW620, but the percentage of CD133 positive cells in SW620 is much higher than those in Colo205 (60% vs. 7%). These data suggest that it is unlikely that CD133 alone can be a useful marker for CRCs, at least not for all types of colon cancer cells.

Subsequently, the CD44/CD133 co-expression profiles of these CRCs were analyzed by flow cytometry. The co-expression of CD44^+^CD133^+^ cells represented a smaller percentage, especially in SW480 (0.1%) and SW620 (2.4%) cell lines (Fig. 2); but it is still very high in DLD1, HCT116 and HT29 cell lines. Thus, these data indicate that the co-expression of CD44 and CD133 was not a minority of CSC subpopulation for half of cells we tested. However, CD44^+^CD133^−^ cells represent a minority in four of six cell lines. To examine the possibility that the combination of CD44 and CD133 can still represent the marker for some colon cancer stem cells, the SW620 cell line, which showed four defined subpopulations for CD44 and CD133 expression, was selected for further characterization. The SW620 line is a metastatic counterpart of the non-metastatic SW480 line, and both cell lines were derived from a colon carcinoma of the same patient (19). SW620 cells were sorted into four subpopulations (CD44^+^CD133^+^, CD44^+^CD133^−^, CD44^−^CD133^+^ and CD44^−^CD133^−^). Their capability of colony formation, proliferation rate, spontaneous apoptosis, drug resistance, as well as their migratory and invasion rates were analyzed and compared.

For colony formation, as shown in Fig. 4, the double positive lines had the highest and double negative the lowest capability while the pair of CD44 positive subpopulations had the greater colony capability compared to the double negative pair. The similar pattern was also observed in the proliferation assay (Fig. 4B). These results suggest CD44 expression plays a major role in the CRC colony formation and proliferation. The data are supported by a previous study involving 60 patients, in which it was shown that CD44 and CD133 positive cells did not co-localize within colorectal cancer. CD44^+^ cells can effectively form a sphere *in vitro* and initiate a tumor *in vivo*. Knockdown of CD44, but not CD133, prevented colony formation and inhibited tumorigenicity in a xenograft model (8).

In addition to robust cell growth, CD44^+^ cells had less spontaneous apoptosis and were more resistant to drug induced cell death (Fig. 5A). This is consistent with results from breast cancer cells, in which CD44 positive cells were shown to express higher levels of the anti-apoptotic protein Bcl-2 (20). It was reported that CD44 is involved in drug resistance and metastasis in prostate cancer (21). Overall, the mixed cells have less spontaneous apoptosis and SW620 is more resistant to CPT induced apoptosis than SW480 (compare last column in Fig. 5A and B). This is consistent with the data produced using cisplatin (22). *In vitro*, SW620 cells were also more irradiation and chemotherapy resistant (23). The mechanism of CD133^+^ cell resistance to apoptosis is believed to be due to the production of interleukin-4 (IL-4) (24).

Surviving cells from drug treatment show only minor change on CD44 expression, but the CD133 expression fluctuates dependent on the CD44 expression on the same cell (data not shown). Overall, the CD44^+^ cells (both CD44^+^CD133^+^ and CD44^+^CD133^−^ cells) were enriched after drug treatment (Fig. 6). This behavior is different for the colon cancer cell lines HT29 and Caco2, which were reported in CD44 and CD133 reduced after treatment with sodium butyrate (25). It is conceivable that these changes may well be cell line and drug dependent. To date, either CD44^+^CD133^+^ or CD44^+^CD133^−^ SW620 cells show characteristics of CSCs. However, the data showed that CD44^+^CD133^−^ are more migratory and invasive in the Matrigel-based assay. It appears that when CD44 is positive, CD133 plays a negative role, and when CD44 is negative; CD133 plays a positive role (Fig. 7). This finding is consistent with the general role of CD44. The CD44 receptor is well documented to interact with the P-glycoprotein to promote cell migration and invasion in cancer (26) and claimed as the invasive marker of glioma (27). A clinicopathological analysis in 189 consecutive CRC patients showed that CD133 was only detected in 29 tumors (15.3%). There was no difference in the distribution of CD133 expressing cells between the invasive and surface area. It was concluded that CD133 expression does not play a dominant role in CRC migration and invasion (28).

In two other clinical studies, CD44s expression reflected more the aggressiveness of the primary tumor (29) and the depth of invasion (30). In addition, the positive correlation of CD44 with metastasis was also demonstrated in prostate CSCs, which showed that cells with enhanced clonogenic, tumor-initiating and metastatic capacities were enriched in the CD44^+^ cell population. CD44 knockdown inhibited prostate cancer regeneration and metastasis (31). Based on above reports and our present data, CD44 is a relatively more robust marker for colorectal CSC, and CD44^+^CD133^−^ is more likely the CSC maker for SW620 cells. However, since the more invasive SW620 has less CD44^+^ cells than SW480, this suggests that CD44 cannot be the sole determinant of cell migration and invasion.

CD44 was reported as a metastasis suppressor for some prostate cancers, and the expression of the standard form of CD44 decreased during the progression of prostate cancer to a metastatic state (32). CD44 also plays a similar negative role in pancreatic cancer as its progression was accompanied by an almost complete loss of CD44 expression, due to alternative splicing of the CD44 pre-RNA. The extracellular matrix can influence the expression of CD44 isoforms and thereby may facilitate tumor invasion (33). This may also be the case for some CRC, as the transition from SW480 to SW620 preceded with a decrease in standard form of CD44 expression, an isoform of CD44 could be expressed on SW620 cells or SW620 has a CD44 with different post-translational modification (such as the glycosylation).

Overall, there is a wide range of expression of both CD44 and CD133 in CRC cells. The data from expression profiling suggest that CD44 or CD133 alone is likely not enough to establish the identity of the CSCs for CRCs. The combination of CD44 and CD133 correlated with more features of CSCs, but they cannot be generalized and applied for all colon cancer cells. CSC surface markers vary depending on the individual source and the stage of colon cancers. For SW620 cells, CD44^+^CD133^−^ correlated with most of features proposed for CSCs, such as minority, drug resistance and invasion capability. Coincidentally, the majority of SW480 cells, the non-metastatic precursor cells of SW620, have the CD44^+^CD133^−^ surface marker, therefore, CD44^+^ and CD133^−^ are not the only determinants of CSCs for these cancer cells. It is possible that there are additional CSC surface markers involved in maintaining CSC properties, or alternatively, the CSC hypothesis may not be a universal model that can be applied to all cancers or all patients with the same disease.

## Figures and Tables

**Table I:** Expression of CD133^+^, CD44^+^ and ESA^+^ in colon cancer cells.

	Colo205	DLD1	HT29	HCT116	SW480	SW620
CD133^+^ cells (%)	6.90± 0.11	12.0±0.38	98.30±0.20	69.62±1.51	0.23±0.06	59.63±1.40
CD44^+^ cells (%)	1.63±0.23	94.0±0.21	91.1±0.20	16.4±0.69	95.8±0.17	3.22±0.63
ESA^+^ cells (%)	99.93±0.12	99.97±0.06	99.97±0.06	99.87±0.05	99.97±0.06	99.97±0.06

## Abbreviations

CRC: colorectal cancer
CSCs: cancer stem cells
ESA: epithelial specific antigen
ALDH1: aldehyde dehydrogenase 1
FACS: fluorescence activated cell sorting
CPT: camptothecin. SDS-PAGE, sodium dodecyl sulfate polyacrylamide gel electrophoresis
PVDF: polyvinylidene difluoride membranes
HRP: horse- radish peroxidase
DAPI: 4′,6-diamidino-2-phenylindole
DMSO: dimethyl sulfoxide
FBS: fetal bovine serum
DMEM: Dulbecco’s modified Eagle’s medium
BSA: bovine serum albumin
